# Asymptomatic Rhabdomyolysis in a Young Adult With COVID-19

**DOI:** 10.7759/cureus.18039

**Published:** 2021-09-17

**Authors:** Abdelhadi Farouji, Rabea Hellou, Asaf Peretz

**Affiliations:** 1 Internal Medicine, Assuta Ashdod Medical Center, Ben-Gurion University of the Negev, Ashdod, ISR

**Keywords:** elevated ck levels, covid 19, rhabdomyolysis, atypical presentation, young adult male

## Abstract

The novel coronavirus disease 2019 (COVID-19) is an ongoing pandemic. Although COVID-19 is frequently associated with respiratory symptoms and complications, multiple extrapulmonary manifestations have been identified since the beginning of the pandemic. Rhabdomyolysis has been described in the literature as one of the extrapulmonary manifestations of COVID-19. Herein, we describe a 21-year-old male patient who presented with cough and fever secondary to COVID-19 confirmed by positive reverse-transcription polymerase chain reaction (RT-PCR) for severe acute respiratory syndrome coronavirus 2 (SARS-CoV-2). The patient presented with an extremely elevated creatinine kinase (CK) of 53,886 U/L (normal 10-170) without any classical symptoms of rhabdomyolysis or deterioration in his kidney function. He was successfully managed with aggressive intravenous fluids. The aim of reporting this case is to highlight the importance of including total CK in the initial evaluation of COVID-19 patients.

## Introduction

Severe acute respiratory syndrome coronavirus 2 (SARS-CoV-2) first emerged in China in December 2019 and shortly thereafter has spread worldwide. According to the Johns Hopkins Coronavirus Resource Center, as of July 24, 2021, more than 193 million patients and 4.1 million deaths have been reported worldwide [1]. The disease may present with different symptoms including cough, fever, myalgia, headache, dyspnea, sore throat, diarrhea, loss of smell or taste, and others [2]. Although respiratory symptoms predominate, different extrapulmonary manifestations have been reported, such as myocardial infarction, myocarditis, pericarditis, pulmonary embolism, stroke, encephalitis, Guillain‐Barre syndrome, and rhabdomyolysis [3]. Herein, we describe a young adult patient who developed coronavirus disease 2019 (COVID-19)-associated rhabdomyolysis without its classical symptoms and signs like myalgia, weakness, or dark urine.

## Case presentation

A 21-year-old patient, with no significant past medical history presented to the outpatient clinic with fever and cough and was diagnosed with COVID-19, confirmed by RT-PCR test. Seven days later, he presented to the emergency room with complaints of fever, cough, and shortness of breath. He denied weakness, myalgia, or any change in the color of his urine. His initial vitals upon arrival revealed a respiratory rate of 18 breaths/minute, heart rate of 95 beats/minute, blood pressure 119/77 mmHg, with a temperature of 39.2 degrees Celsius. The patient was not hypoxic at presentation with oxygen saturation of 98% on room air. His physical examination was unremarkable.

**Figure 1:**
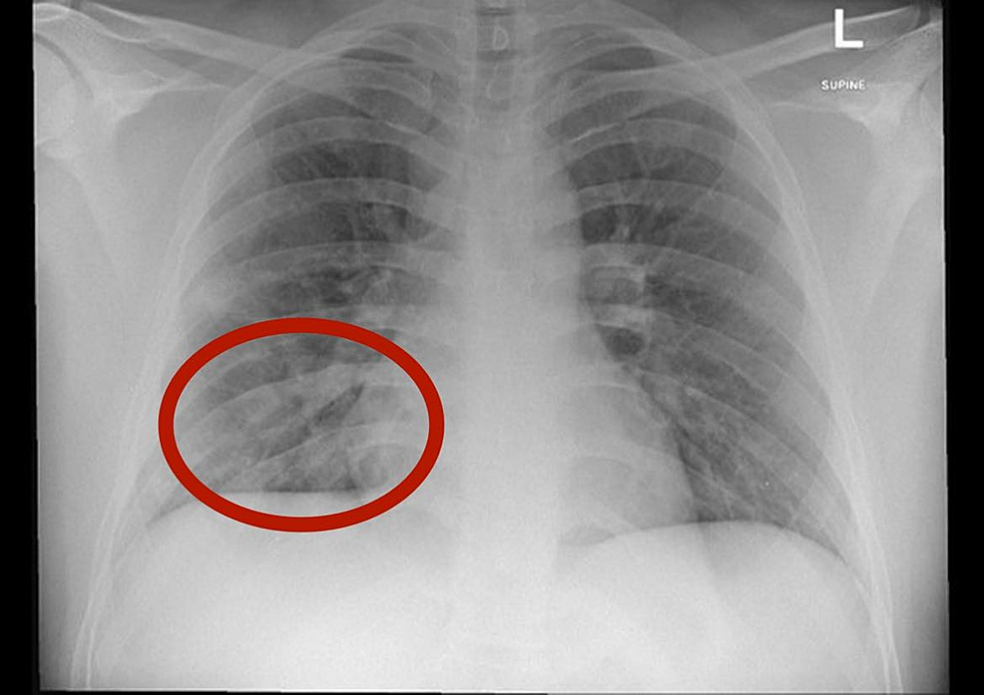
Chest X-ray showing right lobe infiltrates (circle).

His initial complete blood count and metabolic panel were within normal limits. He had total creatinine kinase (CK) of 53,886 units/L (normal 10-170) at presentation to the ER (seven days after diagnosis with COVID-19), creatinine 0.8 mg/dL (normal 0.7-1.2), C-reactive protein (CRP) 35.3 mg/L (normal < 5.00), ferritin 314 ng/mL (normal 30-400), aspartate aminotransferase (AST) 653 units/L (normal 5-40), and alanine aminotransferase (ALT) 182 units/L (normal 4-41). The urine drug screen was negative. Urinalysis and urine myoglobin were not tested. Chest x-ray (CXR) showed patchy infiltrates in the right lower hemithorax (Figure 1).

**Figure 2:**
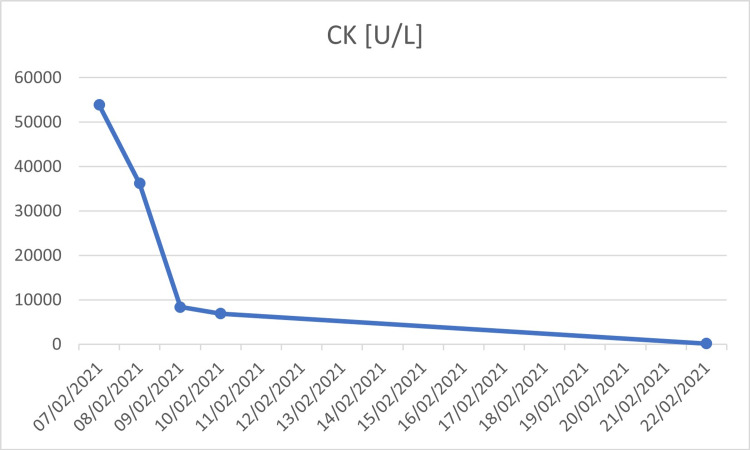
Graph showing trends of CK levels. CK: creatinine kinase

**Figure 3:**
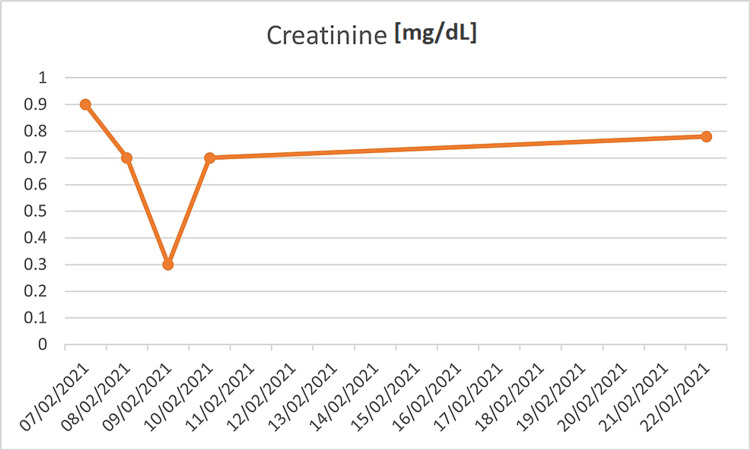
Graph showing trends of creatinine levels.

The patient was admitted to the COVID-19 designated department because of rhabdomyolysis. We excluded other possible causes of rhabdomyolysis; he denied excessive exercise or strenuous activity a few days prior to his admission, no evidence of trauma on physical examination, urine drug screen was negative, ethanol level was undetectable, no past medication history except acetaminophen for fever, nasopharyngeal RT-PCR for the respiratory syncytial virus, influenza A and B were negative, and serology for HIV, hepatitis B and C were negative. This suggests that his rhabdomyolysis was most likely due to COVID-19. During his hospitalization, he received aggressive treatment with an intravenous infusion of normal saline (NaCl 0.9%) at a rate of 200 mL/h. CK levels decreased to 6920 U/L on day four of therapy, following which he was discharged. Twelve days after discharge, blood tests were done at an outpatient clinic and showed a decrease in the CK levels down to 209 U/L. The CK and creatinine levels are shown in the graphs (Figures 2, 3).

## Discussion

Rhabdomyolysis is a potentially life-threatening syndrome characterized by the breakdown of skeletal muscle resulting in the subsequent release of intracellular contents into the circulatory system. These cell contents include enzymes such as CK, glutamic oxaloacetic transaminase, lactate dehydrogenase, aldolase, myoglobin, electrolytes such as potassium and phosphates, and purines [4]. The characteristic complaints in rhabdomyolysis are muscle pain, weakness, and dark urine. However, more than half of the patients may not report muscular symptoms, as was with our patient [5].

Rhabdomyolysis is diagnosed by the elevation in serum CK, and while there is no established serum level cut-off, many clinicians use five times the upper limit of normal (approximately 1000 U/L). Our patient had extremely elevated CK up to 53,886 U/L at the time of admission confirming the diagnosis [6].

Rhabdomyolysis can cause a wide range of complications because of the release of these intracellular contents. One of these complications is acute kidney injury with a reported frequency between 33% and 51%, renal injury can develop when the myoglobin release exceeds the serum protein-binding capacity and the excess pigment precipitates in glomeruli. Other complications include hyperkalemia, metabolic acidosis, disseminated intravascular coagulation, compartment syndrome, arrhythmias, and cardiac arrest [4,5,7].

Many conditions may lead to rhabdomyolysis, not limited to alcohol and drug abuse, medications, trauma, epileptic seizures, excessive exercise, prolonged immobilization, metabolic disturbance, local or generalized muscle ischemia, and heat stroke [8]. Besides that, many bacterial, viral, fungal, and parasitic infections have been described in the literature as causes of rhabdomyolysis [9-11]. Viruses that have been reported to cause rhabdomyolysis are influenza, parainfluenza, coxsackievirus, Epstein-Barr virus, cytomegalovirus, herpes simplex virus, human immunodeficiency virus, adenovirus, echovirus, and hepatitis B and C [12-14]. Influenza virus is the most common cause of viral-induced rhabdomyolysis accounting for approximately 33% of known cases [15].

Since the outbreak of the COVID-19 pandemic, multiple case reports have described COVID-19 associated rhabdomyolysis. Some of these cases described rhabdomyolysis as a potential late complication associated with COVID-19 [16]. Rhabdomyolysis was reported with the initial presentation of COVID-19 infection by others [17-19]. Our case reports a young adult patient that presented with a cough and fever. The unique aspect of our case was the lack of classical symptoms of rhabdomyolysis such as myalgia, weakness, and dark urine with an extremely elevated level of CK up to 53,886 U/L. This suggests COVID-19 to be the likely etiology as the other causes have been ruled out. There are cases that reported COVID-19 associated rhabdomyolysis in pediatric and young adults (18-35 years) (Table 1) [20-25].

**Table 1:** Clinical characteristics of patients (pediatrics and young adults 18-35 years) with COVID-19 associated rhabdomyolysis. M: male; F: female; ADHD: attention-deficit/hyperactivity disorder; OSA: obstructive sleep apnea; HTN: hypertension; SOB: shortness of breath; CK: creatine kinase; COVID-19: coronavirus disease 2019

Author characteristic	Our case	Meegada et al. [20]	Gefen et al. [21]	Tram et al. [22]	McCarthy [23]	Anwar and Al Lawati [24]	Samies et al. [25]
Age (years)	21	19	16	15	34	16	16
Sex	M	M	M	M	M	M	M
Medical history	Healthy	Ulcerative colitis	ADHD, OSA, autism spectrum disorder, morbid obesity, and eczema	Healthy	Prediabetes and obesity	Healthy	Obesity, type 2 diabetes mellitus, OSA, HTN
Trauma or physical exercise	No	No	No	No	Not mentioned	Yes	No
Previous rhabdomyolysis	No	No	No	No	No	Yes	No
Presentation	Cough, fever, and fatigue	Abdominal pain	Myalgia, fatigue, SOB, and cola-colored urine	Proximal muscle pain, tea-colored urine, polyuria, polydipsia, and general fatigue	Fever, cough, SOB, and fever	Fever, sore throat, myalgia, and SOB	Fever, dark-colored urine, myalgia, sore throat, cough
CK on presentation (U/L)	53,886	22,000	427,656	21,876	623	Not mentioned	274,664
Peak CK (U/L)	53,886	22,000	427,656	21,876	5454	Not mentioned	>426,700
Creatinine (mg/dL)	0.9	1.28	0.89	8.91	0.89	Not mentioned	12.03
Treatment	IV fluids	IV fluids	IV fluids and bicarb	IV fluids and bicarb	Not mentioned	Not mentioned	IV fluids, diuretics, and hemodialysis
Outcome	Discharged alive	Discharged alive	Discharged alive	Discharged alive	Died	Died	Discharged alive

The mechanism by which COVID-19 causes muscle damage is uncertain, but various mechanisms have been described for another viral myositis, including direct invasion of muscle tissue by the virus [9,13], immunologic processes induced by the virus that results in muscle damage, and the cytokine storm released in response to viral infection [9,26]. Many cases were reported during the outbreak of SARS, which supports the theory of the cytokine storm theory as the mechanism of the rhabdomyolysis which has been confirmed by the presence of elevated inflammatory markers found in muscle biopsies of patients infected with the virus [27,28].

The treatment of rhabdomyolysis includes aggressive fluid administration, addressing the underlying cause, and management of electrolyte abnormalities in order to prevent acute kidney injury and severe metabolic disturbances [4]. Our patient responded well to the aggressive intravenous fluids; his creatinine level was normal throughout his hospital stay and his CK levels steadily declined. He didn’t develop acute kidney injury or any complications during the course of his disease.

## Conclusions

In conclusion, we are reporting a case of COVID-19-associated rhabdomyolysis in a young adult male patient that developed significant rhabdomyolysis without classical symptoms. Our case aims to highlight the importance of including total CK in the initial evaluation of COVID-19 patients in order to prevent any further complications including acute kidney injury and electrolyte abnormalities.
